# Evaluation of zero-echo-time attenuation correction for integrated PET/MR brain imaging—comparison to head atlas and ^68^Ge-transmission-based attenuation correction

**DOI:** 10.1186/s40658-018-0220-0

**Published:** 2018-10-22

**Authors:** João M. Sousa, Lieuwe Appel, Mathias Engström, Stergios Papadimitriou, Dag Nyholm, Elna-Marie Larsson, Håkan Ahlström, Mark Lubberink

**Affiliations:** 1 Department of Surgical Sciences, Uppsala University, Uppsala, Sweden; 2 Department of Neurosciences, Uppsala University, Uppsala, Sweden; 3 Medical Imaging Centre, Uppsala University Hospital, Uppsala, Sweden; 4 Department of Neurology, Uppsala University Hospital, Uppsala, Sweden; 5 Department of Medical Physics, Uppsala University Hospital, Uppsala, Sweden; 6 MR Applied Science Laboratory, GE Healthcare, Waukesha, USA; 7 PET Centre, Uppsala University Hospital, 75185 Uppsala, Sweden

**Keywords:** Attenuation correction, PET/MR, ZTE-AC, Atlas-AC, Static imaging

## Abstract

**Background:**

MRI does not offer a direct method to obtain attenuation correction maps as its predecessors (stand-alone PET and PET/CT), and bone visualisation is particularly challenging. Recently, zero-echo-time (ZTE) was suggested for MR-based attenuation correction (AC). The aim of this work was to evaluate ZTE- and atlas-AC by comparison to ^68^Ge-transmission scan-based AC.

Nine patients underwent brain PET/MR and stand-alone PET scanning using the dopamine transporter ligand ^11^C-PE2I. For each of them, two AC maps were obtained from the MR images: an atlas-based, obtained from T1-weighted LAVA-FLEX imaging with cortical bone inserted using a CT-based atlas, and an AC map generated from proton-density-weighted ZTE images. Stand-alone PET ^68^Ge-transmission AC map was used as gold standard. PET images were reconstructed using the three AC methods and standardised uptake value (SUV) values for the striatal, limbic and cortical regions, as well as the cerebellum (VOIs) were compared. SUV ratio (SUVR) values normalised for the cerebellum were also assessed. Bias, precision and agreement were calculated; statistical significance was evaluated using Wilcoxon matched-pairs signed-rank test.

**Results:**

Both ZTE- and atlas-AC showed a similar bias of 6–8% in SUV values across the regions. Correlation coefficients with ^68^Ge-AC were consistently high for ZTE-AC (*r* 0.99 for all regions), whereas they were lower for atlas-AC, varying from 0.99 in the striatum to 0.88 in the posterior cortical regions. SUVR showed an overall bias of 2.9 and 0.5% for atlas-AC and ZTE-AC, respectively. Correlations with ^68^Ge-AC were higher for ZTE-AC, varying from 0.99 in the striatum to 0.96 in the limbic regions, compared to atlas-AC (0.99 striatum to 0.77 posterior cortex).

**Conclusions:**

Absolute SUV values showed less variability for ZTE-AC than for atlas-AC when compared to ^68^Ge-AC, but bias was similar for both methods. This bias is largely caused by higher linear attenuation coefficients in atlas- and ZTE-AC image compared to ^68^Ge-images. For SUVR, bias was lower when using ZTE-AC than for atlas-AC. ZTE-AC shows to be a more robust technique than atlas-AC in terms of both intra- and inter-patient variability.

**Electronic supplementary material:**

The online version of this article (10.1186/s40658-018-0220-0) contains supplementary material, which is available to authorized users.

## Background

Attenuation correction (AC) is still a major challenge for PET/MR [1–3], and accurate AC is required for quantitative PET imaging [4]. In stand-alone PET systems, ^68^Ge-transmission scans using rotating rod sources are used for AC [5–7]. Since photon attenuation values are directly measured at 511 keV, this is considered as gold standard in AC [8]. For PET/CT, a low-dose CT scan is acquired for AC, and attenuation coefficients are approximated by conversion from Hounsfield units using well-established methods [5]. PET/MR scanners do not offer a direct method to obtain attenuation maps as the stand-alone PET and PET/CT scanners [2, 7, 9], since MR measures proton density which does not correlate directly to electron density [3, 10]. Furthermore, most MR sequences show little signal in bone and air. Linear attenuation coefficients (cm^−1^) at 511 keV vary between 0 in air, 0.151 in bone and 0.96 in water (6), posing a challenge for MR-based AC. Since ignoring bone may lead to a regional bias, as demonstrated for example by Andersen et al. [11], new methods for MR-derived attenuation maps have been developed and incorporated into the commercially available systems [2, 7].

PET/MR systems have relied on segmentation-based routines for AC, which use 3D fat/water separated imaging for segmentation into four tissue classes (air, lung, soft and adipose tissue), and 511 keV attenuation coefficients are assigned to each tissue class. However, these methods do not consider bone tissue. Therefore, an atlas-based method was introduced using a skull template being co-registered to the individual patient’s data [12]. The reliability of atlas-based AC is associated with how well the template matches the individual patient’s skull. As reported before [13], the atlas method shows good accuracy in the cortex and central parts of the brain, but poor accuracy in the cerebellum when compared to CT-based AC.

A number of other atlas-based methods have been presented as well, for example, the multi-atlas approach by Burgos et al. [14, 15], validated by Sekine et al. [16] and Merida et al. [17], which uses a probabilistic method to synthesise a subject-specific pseudo-CT by registering each atlas to the target subject space. Rezaei et al. [18] proposed a time-of-flight maximum likelihood reconstruction of attenuation and activity (TOF-MLAA) approach, based on the method described by Nuyts et al. [19, 20] to reduce the inaccuracy of MR-AC methods that do not take TOF in consideration. This method was tested by Salomon et al. [21] and Boellaard et al. [22] for improving the accuracy of PET/MR studies using TOF. Keereman et al. [23] proposed using ultrashort echo time (UTE) as a method to visualise the cortical bone and integrate it into MR-based attenuation maps, using a first echo time between 70 and 150 μs. In a recent multi-centre study, Ladefoged et al. [24] compared 11 different MR-AC methods based on the data from a single, non-TOF, integrated PET/MR system (Biograph mMR, Siemens Healthcare, Erlangen, Germany). The first and second echo times for UTE in that paper were 0.07 and 2.46 ms, respectively. It was found that for all AC methods, global radioactivity concentrations are within ± 5% of those derived from CT-based AC.

Recently, a zero-echo-time (ZTE)-based segmentation method [25, 26] has been suggested for eight MR-based AC. ZTE was designed to achieve signal from the cortical bone for tissue segmentation [25–27], and as such can be used to incorporate the bone in MR-based attenuation correction. Sekine et al. [27] assessed the effectiveness and feasibility of ZTE-based AC in a clinical setting compared to CT-based attenuation correction.

The aim of this work was to compare the performance of ZTE- and atlas-based AC using ^68^Ge-transmission scan-based AC as a gold standard. For this purpose, we used the dopamine transporter imaging tracer PE2I in subjects with Parkinsonism. Accuracy and precision were obtained for standardised uptake values (SUV), as well as SUV ratios normalised to the cerebellum, for different clusters of regions across the brain.

## Methods

### Subjects

Ten patients with Parkinsonism (five male, five female; median age 72 years, range 49–82) were recruited by the Department of Neurology at Uppsala University Hospital. Inclusion criteria were (1) at least 40 years of age, (2) a previous clinical ^11^C-PE2I scan on a stand-alone PET scanner, (3) able to undergo an 80-min PET/MR scan and (4) no implants that prohibit MR imaging. One patient was excluded due to technical problems during the image acquisition. This study was approved by the Regional Board of Medical Ethics in Uppsala as well as the Radiation Ethics Committee of Uppsala University Hospital, and all the subjects signed an informed consent prior to inclusion.

### Data acquisition and image reconstruction

A 10-min ^68^Ge-transmission scan using rotating ^68^Ge rod sources was acquired as part of each subject’s prior investigation on a stand-alone PET system (ECAT Exact HR+, Siemens/CTI, Knoxville, TN) [28]. The transmission scan was acquired before tracer administration and hence was free from emission contamination.

PET/MR scans were acquired on a 3 T PET/MR (Signa PET/MR, GE Healthcare, Waukesha, WI), which combines a 3-T MRI with a time-of-flight (TOF)-capable silicone photomultiplier (SiPM)-based PET scanner [29]. Patients were scanned in a supine position using an eight-channel head coil (MR Instruments Inc., Minneapolis, MN, USA). The acquisition protocol was comprised of an 80-min list-mode PET scan starting simultaneously with intravenous administration of 5 MBq/kg ^11^C-PE2I using an infusion pump.

For standard AC, a T1-weighted 3D LAVA Flex was acquired for 18 s with 1 NEX, 500 mm FOV, 5.2 mm slice thickness, matrix 256 × 256 and 5° flip angle. The LAVA Flex sequence generates four image sets (water and fat, in- and out phase) used for MR-AC map generation. Finally, the ZTE acquisition, based on the paradigm proposed by Wiesinger et al. and Delso et al. [25, 26], was acquired for 153 s using 4 NEX, 260 mm FOV, 1.4 mm slice thickness, no slice gap, matrix 192 × 192 and 0.8° flip angle. Finally, a T1-weighted image was acquired for 272 s using a 3D Brain Volume Imaging (BRAVO) sequence with 1 NEX, 250 mm FOV, 1 mm slice thickness, 12° flip angle and a 256 × 256 matrix.

PET images were reconstructed using time-of-flight ordered subsets expectation maximisation (OSEM) with 2 iterations, 28 subsets, a 5-mm Gaussian post-filter, a 192 × 192 reconstruction matrix and a 600-mm FOV. All appropriate corrections, such as for random coincidences and scatter, were applied. Attenuation correction was performed as described in the next section.

### Attenuation correction maps

For each patient, three attenuation correction maps were generated:

- Atlas-based AC
  The atlas-based AC map was generated using a vendor-implemented process comprised of four main steps and taking approximately 30 s with no external interaction [12, 13, 27]. This four-step process consists of (1) T1-weighted image undergoing a Hessian bone enhancement, (2) pseudo-CT generation by rigid and non-rigid B-spline registration of the enhanced image to a CT-based head atlas, (3) generation of an attenuation map from the pseudo-CT using standard energy conversion and resampling and (4) addition of coils and bed to the attenuation map using a CT-based template.
- ZTE-based AC
  This method consists of intensity equalisation [30] followed by logarithmic rescaling to enhance bone tissue and application of a mask to isolate patient data from background and coil elements. The masked image is submitted to a series of threshold operations to define bone and air regions using Gaussian fitting of the main histogram peaks. As a final step, internal air compartments are identified using simple histogram thresholding. The resulting image was co-registered to the patient section of the atlas-AC with coils and bed removed, using rigid-body registration with six degrees of freedom (DOF) and then replaced the patient’s data of the atlas-AC map to create a ZTE-based attenuation map [25, 26]. CT-based template containing coils and bed information was then added again. A more detailed description can be found in Wiesinger et al. [26].
- Transmission-based AC
  Attenuation sinograms were computed by division of the transmission sinogram with a blank scan sinogram, acquired using rotating ^68^Ge rod sources around an empty FOV as part of the daily quality control of the ECAT scanner as implemented in the scanner software. Attenuation maps were reconstructed from the ^68^Ge-transmission data using OSEM with six iterations, eight subsets and a 4-mm Hanning post-filter. A mask was applied to the ^68^Ge attenuation map to remove the bed and head support as well as noise outside the head, and the ^68^Ge attenuation map was co-registered to the head atlas attenuation map with coils and bed removed using six DOF rigid-body registration. Then, the head in the atlas-AC map was replaced by the ^68^Ge-AC map head section. Finally, the coil and bed CT-based template was incorporated into the AC map again. Processing steps were executed using an in-house developed MATLAB pipeline (MATLAB R2015a, Mathworks Inc., Natick, MA).

Using these AC maps, three PET datasets were created, which from here on will be referred to as atlas-AC, ZTE-AC and ^68^Ge-AC.

To evaluate how possible differences between attenuation coefficient values in the different attenuation maps affected the results, atlas- and ZTE-AC maps were rescaled to match ^68^Ge-AC values, and the analysis was repeated. This is described in more detail in Additional file 1.

### Data analysis

For visual assessments, all PET images were spatially normalised using statistical parametric mapping (SPM8; Welcome Trust Centre for Neurological Imaging, London, UK). Then, mean SUV images and mean parametric bias images were generated for ^68^Ge-, ZTE- and atlas-AC.

Atlas-AC, ZTE-AC and ^68^Ge-AC PET images were compared considering uptake values in various brain regions. Agreement between methods was assessed using measures of accuracy, precision and correlations. For this purpose, volumes of interest (VOIs) were placed over the T1-weighted structural MR images using an automated probabilistic VOI template as implemented in the PVElab software [31] and transferred to the co-registered PET images in each dataset. Standardised uptake values (SUV) for each VOI were obtained by retrieving average regional voxel values from all PET images and normalising these to the amount of injected radioactivity per body weight. Then, the SUV values were normalised to cerebellar grey matter to obtain SUV ratio (SUVR) values.

Four clusters of VOIs were created: anterior cortical regions (ACR—cingulate, frontal gyrus), posterior cortical regions (PCR—occipital cortex, parietal cortex and somatosensory motor cortex), striatal regions (STR—caudate nucleus, putamen) and limbic regions (LR—amygdala, hippocampus, hypothalamus and thalamus) [32]. In addition, the whole brain grey matter (WB) and cerebellum (CER) were considered, where the cerebellum is of importance as it is used as a reference region in kinetic analysis for ^11^C-PE2I [32, 33] and many other receptor ligands.

For the comparisons, we used (1) the relative difference in SUV and SUVR between ZTE- or atlas-AC and ^68^Ge-AC (bias; Eq. 1), (2) the standard deviation of the bias (precision) and (3) correlations based on orthogonal regression analysis, assuming equal error variance of the three AC methods.

Bias was calculated as

1 $$ \mathrm{Bias}\left(\%\right)=\frac{\mathrm{SUV}{(r)}_{\mathrm{ZTE}/\mathrm{Atlas}}-\mathrm{SUV}{(r)}_{68\mathrm{Ge}}}{\mathrm{SUV}{(r)}_{68\mathrm{Ge}}}\times 100 $$

Statistical analyses were performed using GraphPad Prism 6 (GraphPad Software, La Jolla, California). A non-parametric Wilcoxon matched-pairs signed-rank test was applied to detect statistically significant differences in bias between AC methods (*p* < 0.05).

## Results

**Fig. 1:**
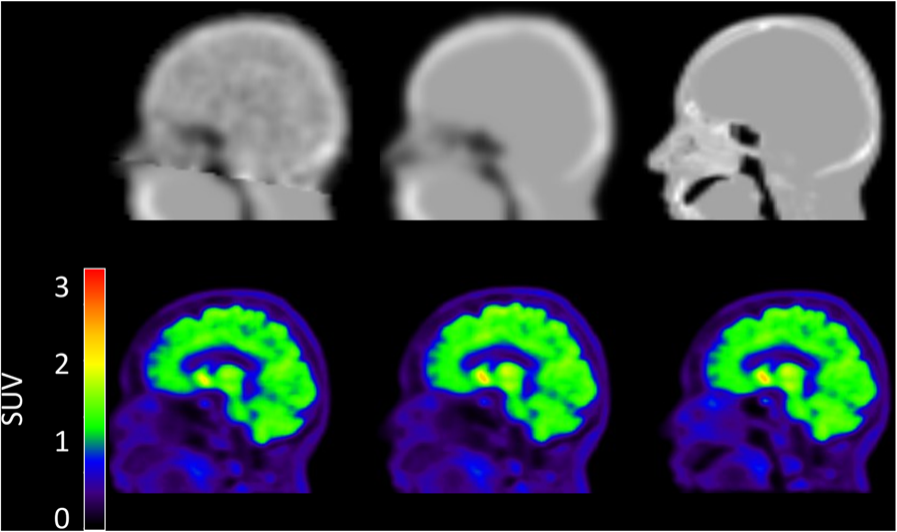
First row, left to right—^68^Ge-, Atlas- and ZTE-attenuation correction (AC) maps; Second row—^11^C-PE2I PET images based on corresponding AC maps expressed as standardised uptake values (SUV)

Figure 1 illustrates ^68^Ge-, atlas- and ZTE-AC maps, as well as corresponding SUV images, for one typical patient with Parkinsonism, showing visually obvious differences between the three AC maps. However, the resulting PET images are visually similar for all cases.

**Fig. 2:**
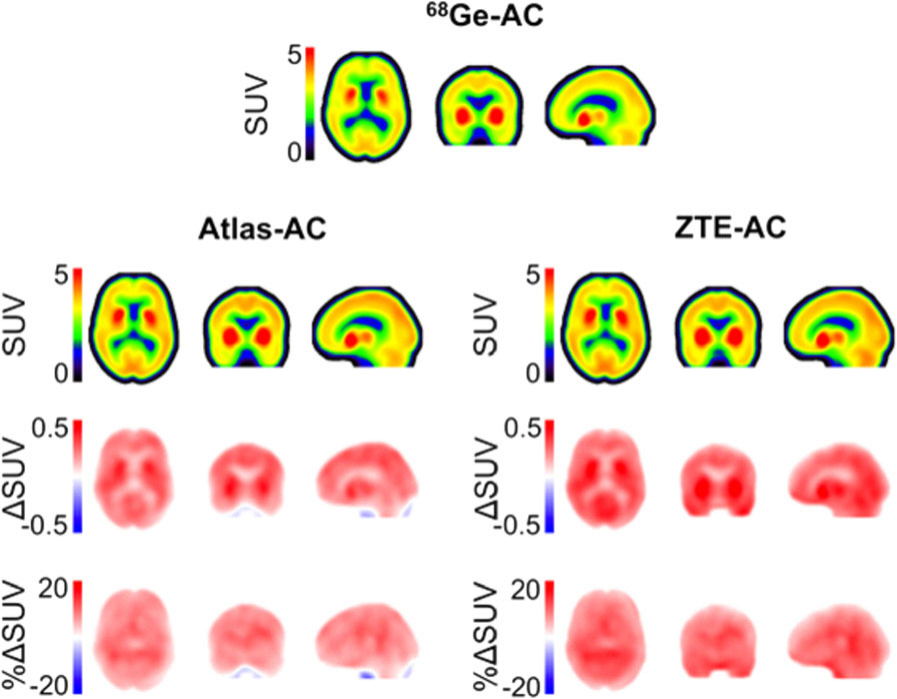
Mean ^11^C-PE2I SUV images using ^68^Ge-AC (first row) as well as atlas-AC and ZTE-AC (second row). Below, average absolute and relative SUV images illustrating absolute (ΔSUV) and relative (%ΔSUV) differences between atlas-AC (left) and ZTE-AC (right) compared to ^68^Ge-AC

Mean ^68^Ge -, atlas- and ZTE-AC SUV images are shown in Fig. 2, demonstrating similar patterns. Mean parametric bias images for ZTE- and atlas-AC compared to ^68^Ge-AC indicated a mostly positive bias in SUV values across the brain for both methods (Fig. 2).

**Fig. 3:**
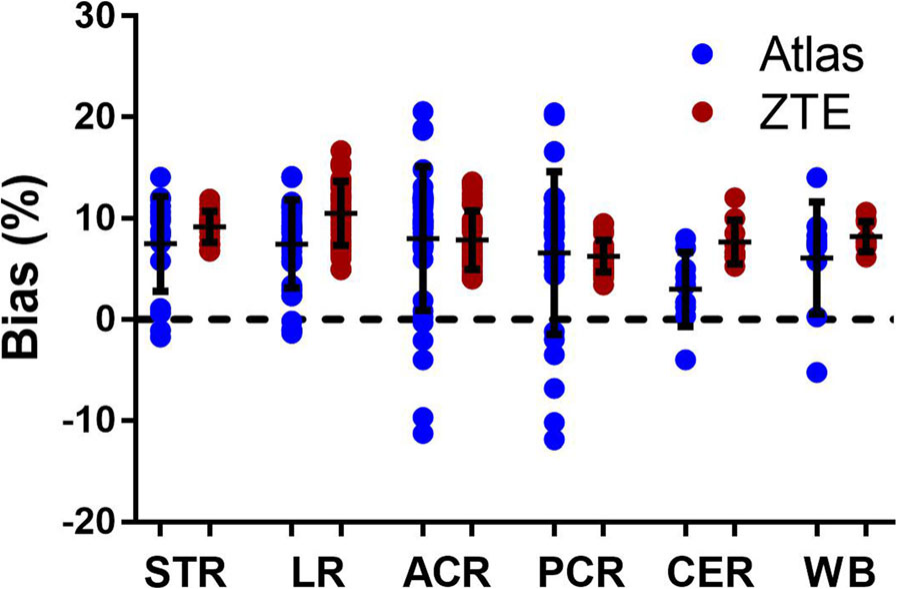
Relative bias in SUV after use of ZTE- or atlas-attenuation correction (AC) compared to ^68^Ge-AC for different regions of interest. STR, striatum; LR, limbic regions; ACR, anterior cortical regions; PCR, posterior cortical regions; CER, cerebellum; WB, whole-brain. Bars and whiskers are mean ± SD

Biases in SUV for ^68^Ge - and atlas-AC compared to ZTE-AC are given for different brain regions in Fig. 3. Both ZTE- and atlas-AC resulted in an overall overestimation of SUV values. Inter-subject variability was larger for atlas-AC than ZTE-AC. Atlas-AC showed a larger inter-subject variability in the cortical regions compared to the striatal and limbic regions.

Quantitative measures of bias in SUV are given for various brain regions in Table 1. Bias was similar for atlas- and ZTE-AC in the cortical regions, about 6–8%. For both CER and LR, the bias is considerably higher in ZTE-AC (8–11%) than for atlas-AC (3–5%). As an overall measure, WB SUV values were slightly higher in ZTE-AC (8.2%) than in atlas-AC (6.1%). The corresponding average SD of the bias was considerably smaller for ZTE-AC (1.5–3.2%) than for atlas-AC (3.7–8.1%). As shown in Additional file 1, this bias is considerably reduced for both methods when rescaling the AC maps to mean ^68^Ge-AC map values.

**Table 1:** Correlation coefficient *r*, slope and intercept of orthogonal regression across subjects for SUV values in different brain clusters when comparing ZTE- and atlas-attenuation correction (AC) with ^68^Ge-AC. In addition, mean bias (%) and accuracy (SD of % bias) are given

AC	Brain region	r	Slope	Intercept	% bias	SD
ZTE	STR	0.99	1.06	0.09	9.2*	1.6
	LR	0.99	1.07	0.06	10.5*	3.2
	ACR	0.99	1.09	− 0.01	7.9*	2.9
	PCR	0.99	1.04	0.04	6.3*	1.6
	CER	0.99	1.03	0.08	7.7*	2.2
	WB	0.99	1.04	0.06	8.2*	1.5
Atlas	STR	0.99	0.97	0.27	7.5*	4.7
	LR	0.97	1.03	0.07	4.5*	4.4
	ACR	0.92	1.06	0.02	8.0*	7.1
	PCR	0.88	1.03	0.05	6.6*	8.1
	CER	0.97	0.92	0.17	3.0	3.7
	WB	0.94	0.99	0.10	6.1*	5.6

*STR* striatum, *LR* limbic regions, *ACR* anterior cortical regions, *PCR* posterior cortical regions, *CER* cerebellum, *WB* whole-brain

*p value < 0.05

**Fig. 4:**
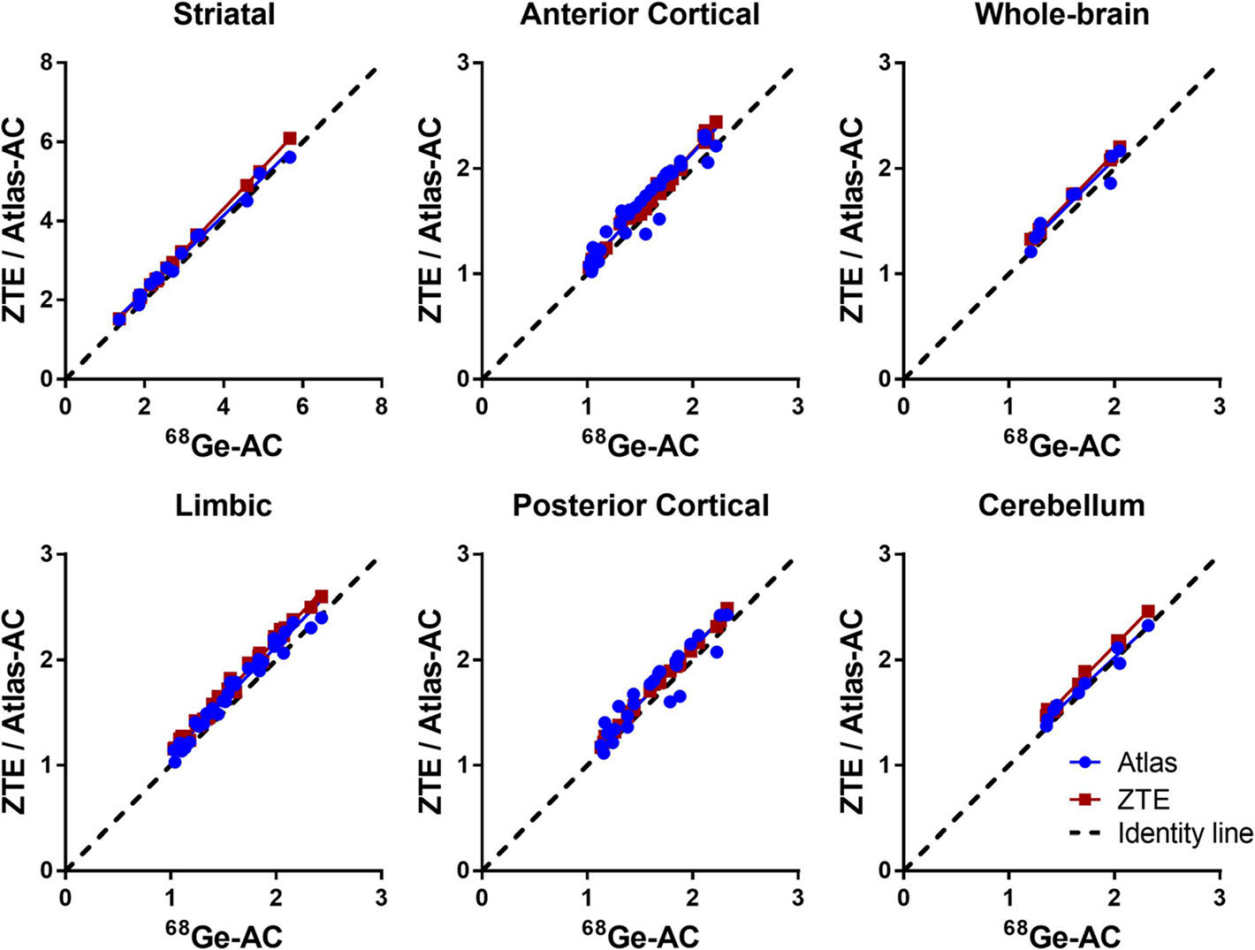
Relationship between SUV for different brain regions when using ^68^Ge-attenuation correction (AC; gold standard) and ZTE- or atlas-AC. Solid lines represent Deming regressions, and the dashed lines represent identity

The relationship in SUV between ^68^Ge-AC and ZTE-/atlas-AC is demonstrated for various brain regions in Fig. 4. Correlation coefficient values were close to 1 for ZTE-AC (0.99) for all regions, whereas *r* values for atlas-AC were still high but more variable (0.88–0.99) Table 1. The slopes of the regression lines were slightly higher than identity across all brain regions for ZTE-AC (1.03–1.09). In contrast, atlas-AC slopes were both lower and higher than the identity (0.92–1.06), depending on the region.

**Fig. 5:**
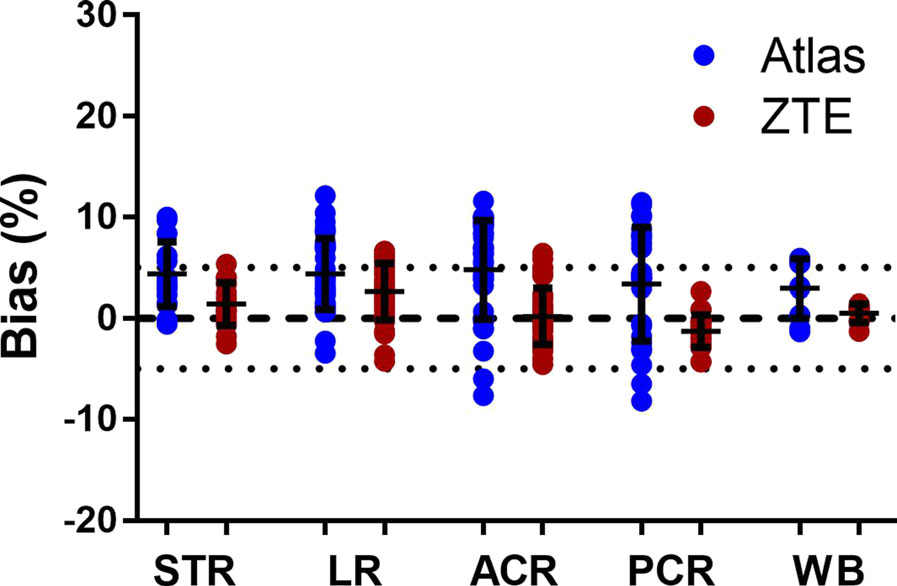
Relative bias in SUVR after use of ZTE- or atlas-attenuation correction (AC) compared to ^68^Ge-AC for different regions of interest. STR, striatum; LR, limbic regions; ACR, anterior cortical regions; PCR, posterior cortical regions; WB, whole-brain. Bars and whiskers are mean ± SD

In Fig. 5, bias in SUVR is given for ZTE- and atlas-AC compared to ^68^Ge-AC considering the different brain regions. Both ZTE- and atlas-AC resulted in a significant overestimation of SUVR values, except in PCR for ZTE-AC. Again, inter-subject variation was larger for atlas-AC compared to ZTE-AC, where the largest variability was found in the cortical regions when using atlas-AC. For SUVR, relative bias and its SD were smaller for ZTE-AC than for atlas-AC for all brain regions (Table 2), especially for the cortical regions (ACR and PCR). Bias in SUVR values varied between 2.9 and 4.8% for atlas-AC and between − 1.3 and 2.6% for ZTE-AC across the regions. The average SD of the bias was considerably smaller for ZTE-AC (0.9–2.8%) than for atlas-AC (2.9–5.6%).

**Table 2:** Correlation coefficient *r*, slope and intercept of orthogonal regression across subjects for SUVR values in different brain clusters when comparing ZTE- and atlas-attenuation correction (AC) with ^68^Ge-AC. In addition, mean bias (%) and accuracy (SD of % bias) are given

AC	Brain region	*r*	Slope	Intercept	% bias	SD
ZTE	STR	0.99	1.00	0.02	1.4*	2.1
	LR	0.96	0.94	0.08	2.6*	2.8
	ACR	0.97	1.14	− 0.12	0.2	2.8
	PCR	0.97	1.01	− 0.02	− 1.3*	1.6
	WB	0.94	0.97	0.03	0.5	0.9
Atlas	STR	0.99	1.00	0.07	4.4*	3.2
	LR	0.94	0.94	− 0.01	4.4*	3.5
	ACR	0.92	1.23	− 0.16	4.8*	4.8
	PCR	0.77	1.21	− 0.17	3.4*	5.6
	WB	0.68	1.41	− 0.35	2.9	2.9

*STR* striatum, *LR* limbic regions, *ACR* anterior cortical regions, *PCR* posterior cortical regions, *WB* whole-brain

*p value < 0.05

**Fig. 6:**
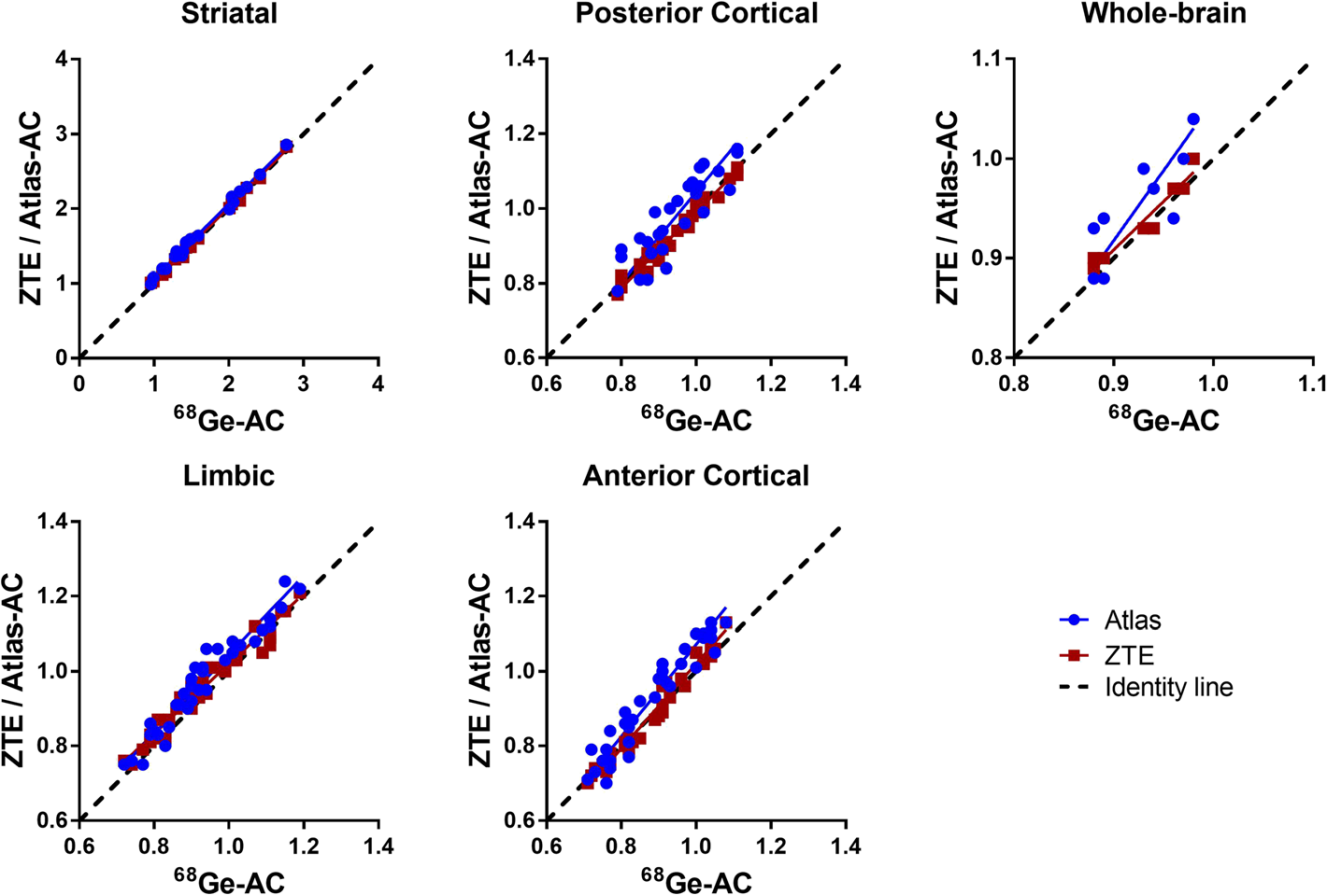
Relationship between SUVR for different brain regions when using ^68^Ge-attenuation correction (AC; gold standard) and ZTE-or atlas-AC. Solid lines represent Deming regressions, and the dashed lines represent identity

In Fig. 6, the relationship in SUVR between ^68^Ge-AC and ZTE-/atlas-AC is illustrated for various brain regions. Correlation coefficients were higher for ZTE-AC (0.94 WB, 0.99 STR) than for atlas-AC (0.68 WB, 0.99 STR), Table 2. The slopes of the regression curves were similar in the subcortical regions (STR, LR) for both AC methods. However, the slopes differed considerably from unity in the cortical regions (1.21–1.23) and WB (1.41) for atlas-AC, but not for ZTE-AC.

## Discussion

In the present work, the accuracy and precision of an MR-AC method based on a proton-density-weighted ZTE sequence were compared to an atlas-based method and the gold standard for PET attenuation correction—the transmission scan with rotating ^68^Ge rod sources [8]. In contrast, other reports [12, 13, 27, 34–39] on MR-AC were all using ^18^F-FDG PET and using CT-based AC as a reference. Two challenges when using ^68^Ge-transmission scans are that they are prone to noise and can suffer from emission spill-over. In the present work, emission spillover was avoided since all transmission scans were acquired before injecting the subjects with ^11^C-PE2I [40].

ZTE-AC presented smaller variability and hence higher precision than atlas-AC. The overall bias in SUV for both atlas-AC and ZTE-AC is probably to a large extent caused by higher linear attenuation coefficients in both ZTE and atlas AC maps compared to ^68^Ge-AC map. SUVR analysis from all brain regions and whole brain showed smaller inter-patient variation and higher accuracy for ZTE-AC compared to atlas-AC. In addition, correlation and agreement between ^68^Ge-AC SUV and ZTE-/atlas-AC were constantly higher for ZTE- than for atlas-AC. Although the accuracy of atlas-AC SUV was better than that of ZTE-AC, showing a smaller mean bias compared to ^68^Ge-AC, the precision of SUV and both accuracy and precision of SUVR were better for ZTE-AC than for atlas-AC when compared to ^68^Ge-AC. A better precision corresponds to a smaller variability in bias and hence more robust and predictable results.

The improvements on SUV data resulting from the use of ZTE-AC are visible as a higher correlation and improved precision compared to ^68^Ge-AC in the cerebellum and PCR (Figs. 3 and 4; Table 1). Similar improvements in the cerebellum have been reported before and can be attributed to improved estimation of the temporal bone [27, 39]. As previously reported [11, 27, 38], misclassification and regular bias using atlas-AC are caused by the slim temporal bone, which may lead to a bias of uptake values in axial slices with lines of response crossing the temporal bone.

**Fig. 7:**
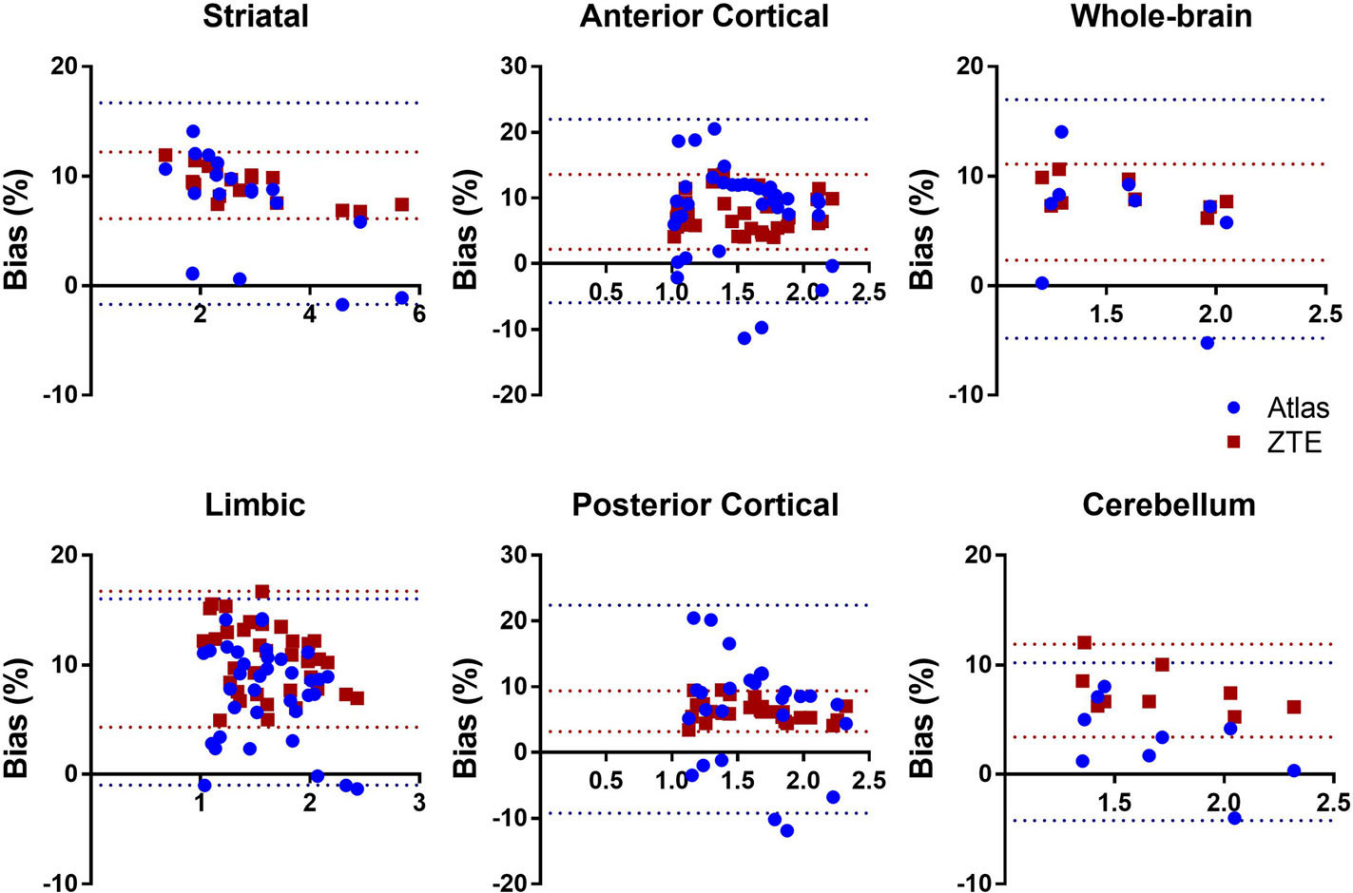
SUV bias plots of ZTE-AC and atlas-AC compared to ^68^Ge-AC for different brain regions. Dashed lines represent confidence intervals associated to ZTE-AC and atlas-AC

**Fig. 8:**
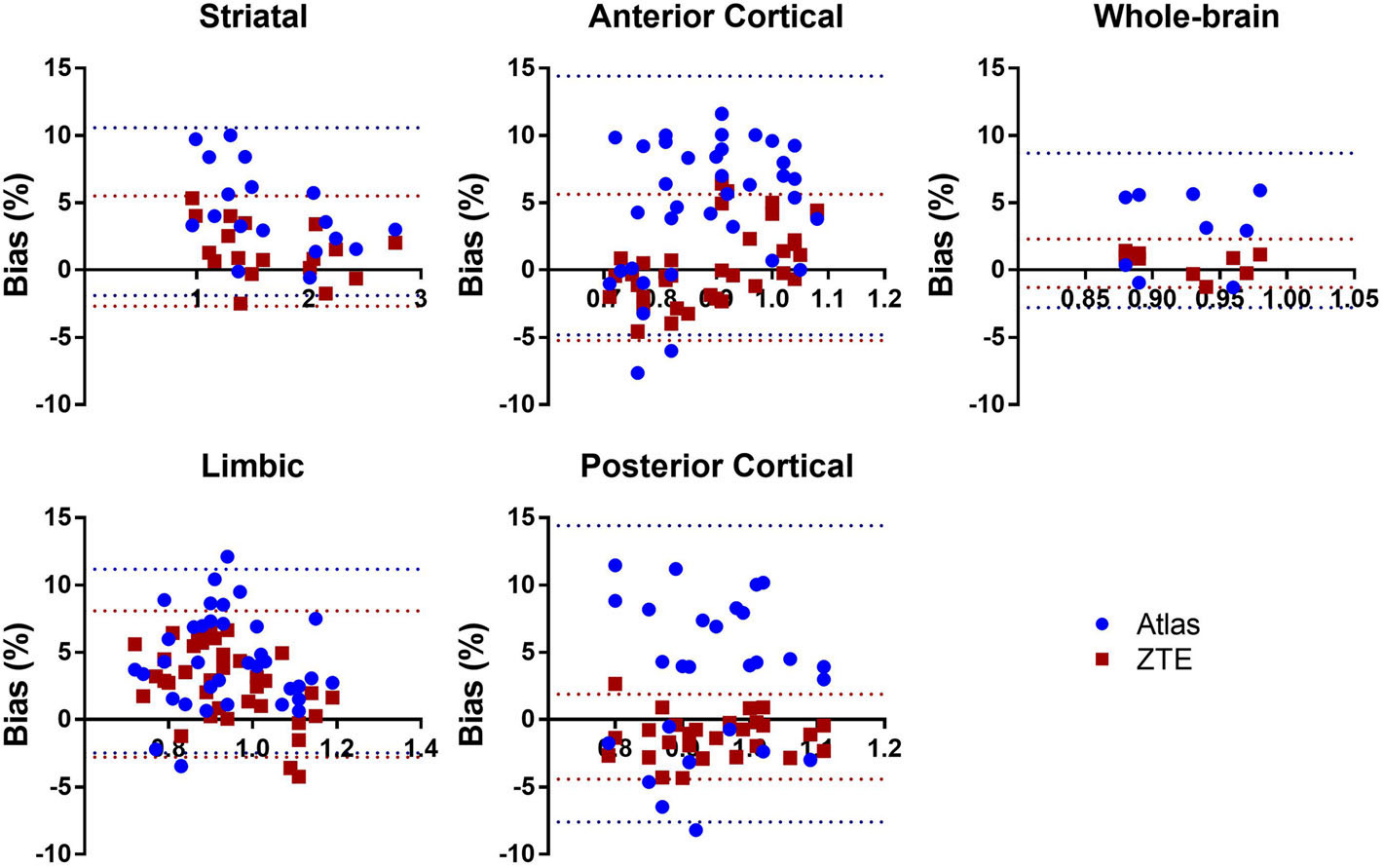
SUVR bias plots of ZTE-AC and atlas-AC compared to ^68^Ge-AC for different brain regions. Dashed lines represent confidence intervals associated to both comparisons

Some misclassifications are visible in the ZTE-AC map (Fig. 1, top right), mostly located around the nasal area, which may lead to cerebellum bias at the level of the sinus. An accurate AC in the cerebellum is of importance since it is used as a reference region for normalisation in studies of neuroreceptor and neurotransporter ligands [41]. These misclassifications are likely caused by the non-continuous mapping of ZTE intensities to attenuation coefficients, which affects mainly the soft tissue near tissue-air interfaces [1, 25, 26, 38, 39]. As a further improvement to the method used in the present study, Yang et al. [39] proposed a sinus and air-tissue interface correction for ZTE-AC to deal with this drawback, reducing SUV bias in the cerebellum from 8% in uncorrected ZTE to 0.6% using sinus correction in their work. However, the present work shows that bias in ZTE-AC SUV values in the cerebellum is comparable to the rest of the brain, Fig. 7. This results in a bias in SUVR, normalised to the cerebellum, compared to ^68^Ge-AC, of no more than a few percent, with much less variability than for atlas-AC (Fig. 8). Hence, in this respect, the present ZTE-AC implementation can be considered to perform sufficiently accurate for receptor ligand studies using the cerebellum as a reference tissue.

Previous studies using ^18^F-FDG have reported smaller mean MR-AC biases in SUV, ranging between − 0.1 and 5.6% for the whole brain [34–37, 39], compared to 8.2% (SUV) and 0.5% (SUVR) for ZTE-AC in the present study. Our results are, however, difficult to compare to those results as these previous studies compared PET/MR-AC to CT-AC, sometimes referred to as the ‘silver standard’ for AC [27], instead of ^68^Ge-AC. CT-AC in itself has been reported as having a mean SUV bias of 2–6% when compared to ^68^Ge-AC in whole-body ^18^F-FDG scans [42, 43]. Although, to our knowledge, no direct comparison between CT-AC and ^68^Ge-AC in the brain scans has been reported, a similar bias could be expected when using CT-AC in the brain.

ZTE-AC and atlas-AC were used as developed by the manufacturer and implemented in the commercial reconstruction software and the research toolbox of the Signa PET/MR, respectively, at the time of the study [8], with the ZTE-AC method as used in the present work being scheduled for commercial release in 2018. Recent improvements of atlas-based methods in comparison with the method implemented in the scanner, such as the multi-atlas approach by Ninon et al. [14, 15] and the probabilistic atlas method described by Merida et al. [17], might have resulted in more favourable results for atlas-based methods but were outside the scope of this work, as were the evaluation of further developments of ZTE-AC [39]*.* ZTE- and atlas-AC methods used in this study apply an attenuation coefficient of 0.1 cm^−1^ in the brain tissue, as does the CT-AC method used by Sekine et al. [27] and many, if not all, clinical PET/CT scanners. However, the mean value of the brain tissue attenuation coefficient in ^68^Ge-based attenuation maps used in the present work is about 0.097 cm^−1^. To assess whether this 3% discrepancy can explain the differences between ZTE- and atlas-AC SUV compared to ^68^Ge-AC in our results, especially in relation to previous studies comparing to CT-AC, ZTE- and atlas-AC maps were rescaled to mean ^68^Ge-AC values using a weighted rescaling to account for differences in scale factors in the brain tissue and bone. PET images were reconstructed using both scaled ZTE- and atlas-AC and analysed again as described in Additional file 1. This led to much smaller biases between MR-AC and ^68^Ge-AC, as shown in the Additional file 1: Figures S2 and S3), which is more in line with published data comparing to CT-AC [1, 13, 27, 34–36, 39, 44]. Also, after rescaling, ZTE-AC did not provide smaller biases than atlas-AC when compared to ^68^Ge-AC, but ZTE-AC did have a considerably better accuracy, similar as when comparing the original ZTE-AC and atlas-AC images to ^68^Ge-AC. The full rescaling results can be found in Additional file 1.

One of the limitations of the present work is that the Signa PET/MR scanner has a greater axial FOV than the ECAT scanner [28]. Therefore, the part of the PET/MR FOV that was not covered by the ^68^Ge-AC image used data from the atlas-AC map. Consequently, the lines of response associated with regions completed by this method may have suffered from a bias towards atlas-AC. However, this effect should be more of an issue for regions below the brain than inside the brain. A 60-cm transaxial FOV with 192 × 192 matrix was used, which results in larger pixel sizes than routinely used in brain studies. Given that rather large regions were used in the analysis, this is unlikely to affect our conclusions. Furthermore, a limited number of subjects (*N* = 9) was included, none of which presented any skull anomalies. The inclusion of patients with missing bone or otherwise abnormal skull would probably have resulted in much larger differences between atlas- and ZTE-AC but were outside the scope of the present work.

Finally, an evaluation of kinetic parameters based on the dynamic PET, such as binding potential and relative cerebellar blood flow, should be assessed to properly understand the use of ZTE-AC for quantitative brain-PET and will be subject of future work.

## Conclusions

The results of the present study show that ZTE-AC is a more robust technique than the clinically implemented atlas-AC method in terms of both inter- and intra-patient variability. ZTE- and atlas-AC had a similar bias in SUV compared with ^68^Ge-AC. The bias in SUV is largely caused by higher linear attenuation coefficients in both ZTE- and atlas-AC maps compared to ^68^Ge-AC map. However, ZTE-AC presented smaller variability and hence higher precision than atlas-AC. SUVR analysis also showed smaller inter-patient variation in addition to higher accuracy for ZTE-AC compared to atlas-AC. ZTE-based AC for quantitative PET brain studies provides a major improvement in comparison to the currently implemented atlas-based method.

## Additional file

Additional file 1: Effects of rescaling atlas and ZTE-AC maps to ^68^Ge-AC values. (DOCX 336 kb)

## Abbreviations

3D: Three-dimensional
AC: Attenuation correction
CT: Computer tomography
DOF: Degree(s) of freedom
FDG: Fluorodeoxyglucose
FOV: Field-of-view
MLAA: Maximum likelihood estimation of activity and attenuation
MR: Magnetic resonance
MRI: Magnetic resonance imaging
NEX: Number of excitations
OSEM: Ordered subset expectation maximisation
PE2I: *N*-(3-iodopro-2E-enyl)-2beta-carbomethoxy-3beta-(4′-methylphenyl) nortropane
PET: Positron emitting tomography
SUV: Standardised uptake value
SUVr: Standardised uptake value ratio
TOF: Time-of-flight
UTE: Ultrashort echo time
ZTE: Zero echo time
