# Supplementary material for: Evaluation of zero-echo-time attenuation correction for integrated PET/MR brain imaging—comparison to head atlas and 68Ge-transmission-based attenuation correction
Source: EJNMMI Phys. 2018 Oct 22;5:20. doi: 10.1186/s40658-018-0220-0 (PMC6196145; doi:10.1186/s40658-018-0220-0)
Supplement: Supplementary file 1 — Effects of rescaling atlas and ZTE-AC maps to 68Ge-AC values. (DOCX 336 kb) [file 40658_2018_220_MOESM1_ESM.docx]

# **Supplemental data – rescaling of AC maps**

## ***Methods***

### ***Attenuation correction maps***

Two masks were created: a brain tissue mask, including all grey and white matter voxels based on the SPM segmentation in PVElab; and a skull mask, created by segmentation of the ZTE-AC maps for each patient. Mean brain and skull AC values were calculated for each AC method and shown in Table S1.

ZTE-and atlas-AC maps were than rescaled so that all voxels within brain and bone masks matched the mean value for ^68^Ge-AC, with intermediate voxels scaled according to the linear interpolation shown in Figure S1.

### ***Data Analysis***

All datasets were analysed as described in the Materials and Methods section.

## ***Results***

The overall SUV overestimation reported in the main Results section was reduced from 8.2 to -0.9% in ZTE-AC and from 6.1 to 0.3% in atlas-AC for WB. SUV ZTE-AC bias, independently of the sign, ranged from around 0.5 to 3% across patients while for Atlas-AC it ranged from 0.2 to 2.5%. The average SD of the bias was considerable smaller for ZTE-AC (1.4 to 2.8%) than for atlas-AC (3.3 to 7.5%), Figure S2. Is worthy of note that the bias associated to cerebellum was reduced from 7.7% to -1.9% in ZTE-AC, while in atlas-AC the bias changed from 3.0% to -2.8%. In all brain region, correlation values (r) were between 0.97 and 1 for ZTE-AC, whereas for Atlas-AC r values, although high, varied somewhat more, (0.94 to 1). The slopes of the regression lines behaved similarly with values close to 1, however ZTE-AC slopes ranged between 0.92 and 1, while Atlas-AC’s from 0.87 to 1, Table S2.

SUVR analysis showed a significant bias for ZTE-AC (0.9 to 3.3%) and Atlas-AC (3.2 to 5.2%) compared to ^68^Ge-AC, except for PCR in ZTE-AC (bias: -0.9%), Figure S3. Inter-subject variability behaved in the same way as for the SUV analysis, with ZTE-AC exhibiting a lower variability than atlas-AC. Both ZTE- and atlas-AC presented high correlation coefficients, with slopes always closer to one for ZTE-AC as seen in Table S3.

***Figure S1****. Rescaling weights for Atlas- and ZTE-AC for several AC values. Left: Atlas-AC weights of 0.966 and 0.964; Right: ZTE-AC weights of 0.966 and 0.851 for brain tissue and skull, respectively. Intermediate values follow the slope of the corresponding curve.*

**Figure S2**. Relative bias in SUV after rescaling of ZTE- and atlas-AC to match ^68^Ge-AC attenuation coefficients, compared to ^68^Ge-AC for different regions of interest. STR=striatum; LR= Limbic regions; ACR=anterior cortical regions; PCR=posterior cortical regions; CER=cerebellum; WB=whole-brain. Bars and whiskers are mean ± SD.

**Figure S3**. Relative bias in SUVR after rescaling of ZTE- and atlas-AC to match ^68^Ge-AC attenuation coefficients, compared to ^68^Ge-AC for different regions of interest. STR=striatum; LR= Limbic regions; ACR=anterior cortical regions; PCR=posterior cortical regions; WB=whole-brain. Bars and whiskers are mean ± SD

***Table S1*** – *Mean AC values in brain tissue and skull for the three different AC methods*

| AC Method | Brain tissue | Skull |
| --- | --- | --- |
| ^68^Ge | 0.0966 ± 0.0001 | 0.111 ± 0.005 |
| Atlas | 0.1 | 0.115 ± 0.009 |
| ZTE | 0.1 | 0.131 ± 0.002 |

***Table S2.*** *Correlation coefficient r, slope, intercept of orthogonal regression across subjects for SUV values in different brain clusters after rescaling ZTE- and atlas-AC to match ^68^Ge-AC attenuation coefficients, with ^68^Ge-AC. In addition, mean bias (%) and accuracy (SD of % bias) are given.*

| AC | Brain region | r | Slope | Intercept | %Bias | SD |
| --- | --- | --- | --- | --- | --- | --- |
| ZTE | STR | 1 | 0.98 | 0.08 | 0.6 | 1.4 |
|  | LR | 0.99 | 0.99 | 0.04 | 1.3* | 2.8 |
|  | ACR | 0.99 | 1.00 | -0.02 | -0.9 | 2.7 |
|  | PCR | 0.99 | 0.96 | 0.02 | -2.7* | 1.8 |
|  | CER | 0.97 | 0.92 | 0.10 | -1.9* | 1.9 |
|  | WB | 0.97 | 0.96 | 0.05 | -0.9 | 1.5 |
| Atlas | STR | 0.99 | 0.92 | 0.24 | 1.3 | 4.3 |
|  | LR | 0.99 | 0.97 | 0.06 | 0.9 | 4.1 |
|  | ACR | 0.96 | 1.00 | 0.04 | 2.3* | 6.5 |
|  | PCR | 0.94 | 0.97 | 0.06 | 1.0 | 7.4 |
|  | CER | 0.98 | 0.87 | 0.17 | -2.8 | 3.4 |
|  | WB | 1 | 0.94 | 0.1 | 0.3 | 5.1 |

* p-value < 0.05.

**Additional footnote:** STR=striatum; LR= Limbic regions; ACR=anterior cortical regions; PCR=posterior cortical regions; CER=cerebellum WB=whole-brain.

***Table S3.*** *Correlation coefficient r, slope, intercept of orthogonal regression across subjects for SUVR values in different brain clusters after rescaling ZTE- and atlas-AC to match ^68^Ge-AC attenuation coefficients, with ^68^Ge-AC. In addition, mean bias (%) and accuracy (SD of % bias) are given.*

| AC | Brain region | r | Slope | Intercept | %Bias | SD |
| --- | --- | --- | --- | --- | --- | --- |
| ZTE | STR | 1 | 1.00 | 0.02 | 2.5* | 1.9 |
|  | LR | 0.98 | 0.95 | 0.08 | 3.3* | 2.8 |
|  | ACR | 0.98 | 1.15 | -0.12 | 1.0* | 2.6 |
|  | PCR | 0.99 | 1.03 | -0.03 | -0.9 | 1.5 |
|  | WB | 0.87 | 1.02 | -0.01 | 0.9* | 0.8 |
| Atlas | STR | 1 | 1.00 | 0.07 | 4.2* | 3.1 |
|  | LR | 0.98 | 1.06 | -0.02 | 3.8* | 3.5 |
|  | ACR | 0.95 | 1.21 | -0.12 | 5.2* | 4.7 |
|  | PCR | 0.88 | 1.19 | -0.15 | 3.8* | 5.4 |
|  | WB | 0.89 | 1.34 | -0.28 | 3.2* | 2.9 |

* p-value < 0.05.

**Additional footnote:** STR=striatum; LR= Limbic regions; ACR=anterior cortical regions; PCR=posterior cortical regions; WB=whole-brain.
